# The contribution of Dutch municipalities to stimulate smoke-free outdoor sports clubs: a qualitative study

**DOI:** 10.1093/heapro/daae011

**Published:** 2024-02-21

**Authors:** Rixt A Smit, Andrea D Rozema, Ien A M van de Goor, Anton E Kunst

**Affiliations:** Department of Public and Occupational Health, Amsterdam UMC, University of Amsterdam, Van der Boechorststraat 7, Amsterdam, The Netherlands; Amsterdam Public Health Research Institute, Van der Boechorststraat 7, Amsterdam, The Netherlands; Tranzo Scientific Center for Care and Wellbeing, Tilburg School of Social and Behavioral Sciences, Tilburg University, Tilburg, The Netherlands; Tranzo Scientific Center for Care and Wellbeing, Tilburg School of Social and Behavioral Sciences, Tilburg University, Tilburg, The Netherlands; Department of Public and Occupational Health, Amsterdam UMC, University of Amsterdam, Van der Boechorststraat 7, Amsterdam, The Netherlands; Amsterdam Public Health Research Institute, Van der Boechorststraat 7, Amsterdam, The Netherlands

**Keywords:** local policy, municipality, smoke free, sports clubs, smoking

## Abstract

Local governments may play a key role in making outdoor sports clubs smoke free. This study aims to assess the activities, motives, challenges and strategies of Dutch municipalities regarding stimulating outdoor sports clubs to become smoke free. Semi-structured interviews were conducted with 19 policy officers of different municipalities in the Netherlands. The included municipalities varied in terms of region, population size and degree of urbanization. Data were analyzed using content analysis. Municipalities stimulated sports clubs to become smoke free by providing information and support and, to a lesser extent, by using financial incentives. Motives of municipalities varied from facilitating a healthy living environment for youth, responding to denormalization of smoking and aligning with goals formulated in national prevention policy. Policy officers faced several challenges, including limited capacity and funds, a reluctance to interfere with sports clubs and little support within the municipal organization. These challenges were addressed by employing various strategies such as embedding smoke-free sports in a broader preventive policy, setting a good example by creating outdoor smoke-free areas around municipal buildings, and collaborating with stakeholders in the municipality to join forces in realizing smoke-free sports clubs. Municipalities demonstrated evident motivation to contribute to a smoke-free sports environment. Currently, most municipalities fulfil an informative and supportive role, while some municipalities still explore their role and position in relation to supporting sports clubs to become smoke free. Other municipalities have established, according to them, effective strategies.

**Contribution to Health Promotion**

- Local governments may play a key role in stimulating outdoor smoke-free environments.
- We identified activities, motives, challenges and strategies of Dutch municipalities in stimulating outdoor sports clubs to become smoke free.
- Most municipalities fulfilled an informative and supportive role, motivated by both internal and external factors.
- Policymakers encountered challenges such as limited capacity and funds, reluctance to interfere with sports clubs and limited municipal support.
- Strategies involved embedding smoke-free sports in preventive policy, creating outdoor smoke-free areas around municipal buildings, and collaborating with multiple stakeholders.

## BACKGROUND

Several countries aspire to realize a smoke-free generation in the near future, that is, a smoking prevalence of 5% or less. This pursuit involves various measures, such as preventing youth from smoking initiation and protecting them from the hazards of second-hand smoke (SHS). In recent years, implementation of smoking bans has extended from indoor public places to outdoor areas. Outdoor smoke-free environments are essential for protecting the health and development of children (WHO, 2021). Implementing smoke-free policies (SFPs) contributes to reducing the exposure of youth to tobacco smoke, which supports the belief that smoking is a rarity rather than normal and common behavior (Alesci, Forster and Blaine, 2003; Wilcox, 2003; Leatherdale *et al*., 2005). As a result, SFPs may reduce the likelihood of smoking initiation among youth (Siegel *et al*., 2008; Shang, 2015).

In the Netherlands, following years of active political lobbying and an extensive campaign by the Smoke-Free Generation movement, the National Prevention Agreement (NPA) was signed by over 70 organizations (Ministry of Health, Welfare and Sport, 2019). This agreement included new national policy measures, such as increasing tobacco taxes, a display ban on tobacco products, and prohibiting smoking on school premises. The NPA also fostered a bottom-up approach by describing actions for smoke-free environments at societal organizations such as sports clubs and children day care centers. Local governments were expected to stimulate and respond to this bottom-up initiative. Municipalities could be influential to sports clubs especially since many clubs receive grants and rent venues from the municipality.

In 2020, 73% of Dutch municipalities indicated that they were involved in realizing a smoke-free generation and 58% stated that they played a role in making outdoor sports clubs smoke free (I&O Research, 2020). Recent research found that outdoor sports clubs are more likely to be smoke free when the municipality has a strong local tobacco control policy (Garritsen, Submitted for publication). However, municipalities differed with regard to the strength of those policies, from none (i.e. municipalities do not play a role) to strong (i.e. municipalities played an activating and/or regulating role).

In Europe, implementation of smoke-free environments by local governments has predominantly focused on indoor areas of youth-related settings (Mlinarić *et al*., 2020), although there are some examples of initiatives by local municipalities to realize smoke-free parks, squares and beaches (WHO, 2014; BBC, 2015; Guardian, 2016; Connexion, 2018). Research on the adoption and implementation processes of local tobacco control policies has mainly been conducted outside Europe (Satterlund *et al*., 2011; Klein *et al*., 2014; Fong *et al*., 2015; Septiono *et al*., 2019). To date, it is unknown how Dutch municipalities see their role in stimulating outdoor sports clubs to become smoke free, and which circumstances may influence the execution of this role. Therefore, the aim of this study was to assess the activities, motives, challenges and strategies of municipalities regarding stimulating outdoor sports clubs to become smoke free.

## METHODS

### Study setting

We conducted semi-structured interviews with policy officers of municipalities throughout the Netherlands. In total, 19 policy officers of different Dutch municipalities were included in this study. The included municipalities varied in terms of region, population size and degree of urbanization (see Table 1).

**Table 1:** Characteristics of the study sample

	*n* = 19	%
Characteristics of respondents		
Gender		
Female	14	73.7
Male	5	26.3
Duration of employment		
1–2 years	8	42.1
2–5 years	7	36.8
>5 years	4	21.1
Characteristics of municipalities		
Strength of local tobacco control policy^a^		
None/weak	2	10.5
Fair	4	21.1
Moderate	4	21.1
Strong	5	26.3
Unknown	4	21.1
Percentage of smoke-free sports clubs^a^		
0–3%	5	26.3
14–25%	4	21.1
26–45%	7	36.8
46–100%	2	10.5
Population size of municipality		
10 000–20 000 people	4	21.1
20 000–50 000 people	5	26.3
50 000–100 000 people	6	31.5
150 000–250 000 people	4	21.1
Degree of urbanization		
Highly urbanized region	2	10.5
Urbanized region	6	31.6
Moderately urbanized region	3	15.8
Rural region	7	36.8
Highly rural region	1	5.3

^a^The data presented are derived from previous analysis; for further information, see Garritsen (submitted for publication).

### Participants

Our recruitment strategy was designed to ensure that each municipality in the Netherlands had an equal opportunity to participate in this study. A flyer was designed describing the rationale of the study, the criteria for participation and the invite for an interview. The flyer did not specify that municipalities had to actively engage in stimulating SFPs in sports clubs. This allowed municipalities with varying levels of involvement in such initiatives to participate in the study and share their experiences regarding the adoption or implementation of SFPs. The flyer stated that people were eligible to participate in the study if they were involved in the development of local tobacco control policies.

The flyer was sent to potential participants using three different channels: (i) by sending it to municipalities via a general mail address or a contact form on their website, (ii) by sharing it in a LinkedIn-post calling for participants and (iii) by asking municipal health services and the Association of Sports and Municipalities (a national umbrella organization representing all municipalities in the field of sports) to distribute the flyer in their networks.

In total, we were able to approach 343 of the 345 municipalities in the Netherlands. We received a response to the flyer from 26 people, all from different municipalities. Five people responded that they were unable to participate due to a lack of time or a short-term employment within the municipality, and two people did not respond after the initial contact was made. Eventually, 19 respondents participated in the study, whose job titles were either policy officer (*n* = 11) or policy advisor (*n* = 8), and who worked in domains called Public Health, Sports, Prevention, and/or Social Affairs. In the Dutch context, policy officers are generally more focused on the implementation of policy, while policy advisors engage in formulating and advising on policy options. However, the exact job roles and responsibilities vary according to the municipality, and terms are used interchangeably. Fourteen of the respondents were female (73.7%), and the mean age was 35.5 years (SD = 7.4). Other characteristics of the respondents and the municipalities are presented in Table 1.

### Procedure

Semi-structured interviews were conducted by the first author (R.A.S.), who is a trained interviewer with experience in qualitative research methods. An interview guide (see Supplementary Appendix I) was developed and partly inspired by the Unified Model of Government Innovation, which can be used to provide insight into why some local governments are faster with adopting certain policies than others (Berry and Berry, 2018). The emphasis of the interview guide was primarily on gaining insight into municipalities’ activities, motives, challenges and strategies regarding stimulating outdoor sports clubs to become smoke free. The interview guide was reviewed by all authors and pilot tested on two individuals who have experience with working within a local government and who were able to provide feedback on the clarity and comprehensibility of the questions. After two thirds of the interviews were completed, we found that almost no new information was emerging from further interviews. All interviews were conducted online, audio recorded and lasted on average 38 min (range 26–49). Respondents signed an informed consent form and received a €25 gift voucher for their participation.

### Ethical approval

The Medical Ethics Review Committee of the Academic Medical Center confirmed that the Dutch Medical Research Involving Human Subjects Act (WMO) did not apply to this study and that an official approval was not required (W20_318 # 20.369).

### Analysis

Interviews were transcribed verbatim by a professional transcript company and analyzed using MAXQDA (Verbi, 2021). Content analysis, a systematic method to analyze qualitative data, was used to interpret meaning from the data (Hsieh and Shannon, 2005). Coding was conducted by the first author (R.A.S.), and a research intern coded five transcripts in parallel to ensure consensus about the codes assigned to the passages. Codes that represented similar information were clustered into subthemes, which were subsequently categorized into four main themes (activities, motives, challenges and strategies). Multiple discussions were held with all authors regarding the clustering of the coded passages into subthemes and overarching themes.

## RESULTS

The results will be discussed in four sections: (i) activities of municipalities to stimulate outdoor sports clubs to become smoke free, (ii) motives for those activities, (iii) challenges that municipalities face in stimulating outdoor sports clubs to become smoke free and (iv) strategies that are used to deal with those challenges.

### Activities of municipalities to stimulate SFPs in outdoor sports clubs

This section will elaborate on two ways in which municipalities stimulated sports clubs to become smoke free: by providing information and support and by using financial incentives.

Respondents mentioned that targeted actions to stimulate SFPs in outdoor sports clubs mainly consisted of providing information and support for clubs. Municipalities tried to reach sports clubs and to disseminate information regarding SFPs via several channels, e.g. distributing a newsletter, organizing inspiration sessions and workshops. Clubs were offered support in various ways. Some municipalities contracted a club support officer who would be available to clubs to answer potential questions and who referred clubs to the right organization when concrete support or advice regarding implementation of SFPs was needed. The latter include local (sports) coaches employed at sports advisory organizations (in Dutch: JOGG-Teamfit or Team Sportservice).

The club support officer is the first point of contact for the sports clubs. It can be about topics that are health related. So if a sports clubs wants to position themselves as a healthy sports club and wants to get started with becoming smoke-free, they can go to the advisor who will support them in this. (Respondent 001)

A few respondents mentioned that their municipality was using financial incentives to stimulate SFPs in sports clubs. Sports clubs could apply for a subsidy to make their entire venue smoke-free and to use standard smoke-free signs that were developed and distributed by the Health Funds for Smoke-Free (Willemsen and Been, 2022). Respondents got the impression that the financial contribution appeared to work as a stimulus for sports clubs to become smoke free.

We are providing grants, which means that sports clubs receive 1000 euros if they make their outdoor area completely smoke-free, including the entrance. Since we did that, we noticed that quite a few sports clubs have become smoke-free*. (*Respondent 014)

### Motives for local activities

According to respondents, municipalities were motivated to aim for a smoke-free sports environment for different reasons. First, municipalities felt a certain responsibility for enabling youth to grow up healthy. They wished to provide a healthy, smoke-free living environment, particularly focusing on places where children are frequently spending their time, such as sports clubs.

We mainly focus on youth and we want them to be able to grow up in smoke-free environments so that they are in less contact with smoking and that they are also less likely to start smoking themselves. That can be at the bus stop, that could be the town hall, but that should also be the sports club. (Respondent 007)

Second, the shift toward non-smoking as a social norm, together with the launch of national initiatives such as the Smoke-Free Generation campaign, led to increased awareness and public support regarding the importance of smoke-free environments. This convinced municipal stakeholders that it was the right time to focus on this matter.

The national attention helps in people being more ready for it and understanding it. It makes sense you know, of course you won’t smoke around children anymore. The time is really here for it. (Respondent 004)

Third, the NPA as concluded by the Dutch government in 2018, called for local authorities to commit themselves to health prevention and to implement concrete policy plans that would contribute to creating a smoke-free generation by 2040.

We have to draw a local prevention agreement according to the national guidelines and smoking is part of that, so we translated that actively, which is why we included it in our agreement. (Respondent 003)

Finally, some respondents used policy plans from other municipalities as inspiration and indicated that they did not want to stay behind.

If they really show that they are going for smoke-free sports, that can of course be a trigger to see if we as a municipality should also do something with that. In that sense, as a municipality, you don’t want to stay behind. (Respondent *001)*

### Challenges that municipalities face in stimulating SFPs in outdoor sports clubs

In this section, we will discuss three challenges that municipalities encountered when stimulating outdoor sports clubs to become smoke free: limited availability of capacity and funds, reluctance to interfere with sports clubs, and little support within the municipal government.

According to respondents, limited capacity and financial resources restrained the approach that municipalities could develop. Respondents indicated that municipalities generally had a small budget, with about one or two policy officers to employ in the Public Health domain. The use of the scarce financial resources depended on the priority that was given to various themes within this domain of a municipality.

We have very little budget, and with that, you must pay for all kinds of prevention, so not just smoking, but also overweight, alcohol, drugs. So, that is very broad, and you really must make conscious choices. (Respondent 005)

Some respondents indicated that municipalities took a cautious approach toward sports clubs. As sports clubs generally exist by the virtue of volunteers, municipalities were reluctant to overburden them or generate resistance by requiring them to become smoke free. Therefore, municipalities refrained from imposing a smoking ban on sports clubs. Instead, they preferred that clubs themselves were intrinsically motivated to adopt an SFP, presuming that this would benefit subsequent SFP implementation.

We are very glad that there is a rich involvement in sports clubs, that people want to volunteer for sports clubs. And you want to maintain that. So, there is a certain reluctance to demand too much from the clubs. (Respondent 001)

Respondents indicated that support for realizing a smoke-free sports environment was required at all levels of the municipal organization. A prime role was played by the municipal council, as a municipal council that supported the ambition of smoke-free sports clubs was expected to accelerate and ease the process. On the contrary, when relevant aldermen resisted such an initiative, it was perceived as challenging to convince them to invest time and resources to support smoke-free sports clubs.

It would be very helpful if the alderman would be passionate about the smoke-free theme, which is not the case now. That means I basically don’t have time for that in my range of duties. While if the board of mayor and aldermen would say: we are putting smoking on the agenda, we can also make time for that. (Respondent 018)

### Strategies of municipalities to deal with potential challenges

This section will elaborate on three strategies that municipalities employ to address potential challenges: setting a good example as municipality itself, embedding smoke-free sports clubs in a broader policy, and working together with various parties in the municipality.

Many respondents indicated that the municipality’s primary step was to set a good example, for example by ensuring that the outside areas of their own municipal buildings were smoke free. In addition, some municipalities also provided smoking cessation services for their own employees. Setting a good example was considered as a crucial step by respondents, as this would increase the credibility of the municipality and their message concerning the importance of smoke-free environments.

At the time, the alderman smoked and in the context of setting a good example, it is not helpful to have him proclaim smoking is not healthy. Then you do not come across as credible. (Respondent 013)

Municipalities often embedded making sports clubs smoke-free within broader policy. Respondents mentioned two policy frameworks. First, smoking prevention and ensuring smoke-free environments for children were often formulated in the context of a local prevention agreement. This agreement included policies aimed to realize smoke-free areas in places frequently visited by children, such as schools, recreation parks and sports clubs. Second, in other municipalities, smoke-free sports initiatives were included in a so-called sports agreement, which aimed for a healthier sports environment through a variety of measures, such as a healthier supply of food and drinks in the sports canteen, specific rules for alcohol consumption and rules to ban smoking from the venue of sports clubs.

In our local prevention agreement, we included stimulants as an extra item and smoking is in principle also a stimulant. In addition, we promote a healthy lifestyle, which also has a lot of overlap with smoking, because smoking is of course very unhealthy. So then we were like: if we want to have a healthy sports club, we must also ensure that there is no smoking, then we have 2 action points at the same time: we want to promote a healthy canteen, but we also want to say: no smoking on site. So that overlaps a lot and that supports each other. (Respondent 017)

Many respondents indicated that their municipality was cooperating with other parties in the municipality to realize smoke-free environments, not only at sports clubs but also at, for example, school premises and child playing grounds. Partnerships were formed with stakeholders such as general practitioners, hospitals, schools, addiction care organizations and municipal health services. The involved parties committed to, among others, realize smoke-free environments. According to respondents, such a collaboration functioned effectively in case that a common goal was shared by various parties. A main motive for such collaboration was that the municipality would not be the only actor to be ‘blamed’ for policies that might be perceived as intrusive.

That also helped, that we as a municipality were not the only one pointing the finger to the sports clubs, but that it came from the entire city. So, it wasn’t just the sports clubs that had to do something, but everyone did their part in this. (Respondent 002)

## DISCUSSION

### Key findings

Dutch municipalities stimulate SFPs in outdoor sports clubs by providing information, support and financial incentives, as they are motivated by the idea of enabling youth to grow up healthy, responding to the denormalization of smoking and aligning with national smoking prevention goals. However, policy officers face several challenges such as limited capacity and funding, a reluctance to interfere with sports clubs and little support within the municipal government. These challenges are dealt with by using strategies such as embedding smoke-free sports in a broader preventive policy, setting a good example by creating outdoor smoke-free areas around municipal buildings, and collaborating with stakeholders in the municipality to join forces regarding stimulating SFPs in outdoor sports clubs.

### Interpretation of findings

The various reasons for municipalities to stimulate outdoor sports clubs to become smoke free are all in accordance with the considerations that sport clubs have themselves for adopting outdoor SFPs: protecting children from exposure to SHS and smoking initiation, responding to a changing societal norm with regard to smoking, and experiencing external pressure to become smoke free (Garritsen *et al.*, 2021, 2022). Municipalities are not only driven by internal motives but also driven by external motives, such as the wish to not stay behind the policies of adjacent municipalities. This may lead to a horizontal diffusion pattern, where municipalities are more inclined to adopt an SFP if their neighboring municipalities do so. Similar diffusion effects have been observed in a study that explored the adoption of SFPs by local governments in Indonesia (Septiono *et al*., 2019).

Commonly, local policy officers took a cautious approach toward sports clubs. They are wary of overburdening clubs, given their dependence on volunteers and their financial vulnerability. A survey in 2023 found that 23% of Dutch outdoor field sports clubs hold a pessimistic outlook on their club’s future, due to a decreasing number of members and volunteers, the rising (energy) costs and decreasing income (Dalhuisen *et al*., 2023). Members of sports clubs expect that adopting an SFP might reduce the number of volunteers, the time people spend at the club and the money they spent at the bar, which would ultimately put the social and financial balance of the club at risk (Garritsen *et al.*, 2021). Sports clubs may show defensive reactions when stringent measures are being imposed upon them (Skille, 2008; Koski, 2012). This highlights the need for municipalities to strike a balance between adopting effective and strong local tobacco control policies, while considering the effect it may have on sports clubs.

A small number of municipalities in our study sample used financial incentives to stimulate sports clubs to become smoke free. While policy officers of those municipalities held a positive view of this approach, it remains unknown whether this is indeed an effective strategy to increase SFPs in sports clubs. A local smoke-free alliance in the UK has incentivized sports clubs to adopt SFPs during youth activities by providing grants of £500, with the effect that 87% of the participating sports clubs adopted and enforced an SFP (Somerset Community Foundation, 2014). While considerable research has been conducted on using financial incentives to influence smoking behavior of individuals (Tappin *et al*., 2015; Notley *et al*., 2019; Pisinger *et al*., 2022), little is known about the effectiveness and best practices of using financial incentives to encourage (smoke-free) policy adoption in non-commercial and/or voluntary organizations such as sport clubs.

The strategies that were suggested by the respondents do not directly align to the challenges that they identified earlier in the interviews. Yet, on further reflection, each of the strategies appears to be relevant to some of the challenges. First, setting a positive example in having smoke-free outdoor areas at municipal buildings may enhance the credibility and strength of the municipality’s position when engaging with sports clubs, which is particularly relevant considering the need for a cautious approach toward sports clubs. Second, embedding smoke-free sports clubs in broader policy may increase the chance of garnering greater support within the local government. If part of a broader health-related policies, spanning various policy areas and domains, it may engage a larger number of policy officials, thereby fostering increased support within the municipality. Third, working together with various local organizations has the potential to alleviate challenges tied to limited capacity and financial resources. When multiple organizations are dedicated to initiatives like creating smoke-free environments, it increase the personal commitment and financial resources needed to stimulate sports clubs to become smoke free.

In the Netherlands, establishing a smoke-free sports environment is currently stimulated via a bottom-up policy approach. Our findings suggest that this approach both motivates and presents challenges for municipalities. On the one hand, municipalities effectively leverage the benefits of the bottom-up approach by collaborating with various local stakeholders, which collectively work toward achieving the Smoke-Free Generation goal. On the other hand, municipalities have a limited budget to address multiple prevention themes, including nutrition and physical activity, which often compete with smoking prevention for limited resources. Additionally, the complex dynamics between sports clubs and municipalities may hinder stimulating widespread adoption of outdoor SFPs at sports clubs. Therefore, top-down approaches such a binding smoke-free legislation may in the long run be needed to ensure SFP adoption by all sports clubs. Bottom-up successes, even though partial, may leverage the popular support needed for such legislation.

### Potential limitations

There are some potential limitations that should be considered when interpreting our findings. First, as we recruited a small part of all Dutch municipalities, the study may be subject to selection bias. Potentially, we have interviewed policy officers who predominantly had positive experiences with local policies to stimulate SFPs in sports clubs. We aimed to avoid this bias by calling for policy officers who were involved with smoking policies, without specifically referring to outdoor sports clubs. Furthermore, our sample is representative of the Netherlands in terms of general characteristics of municipalities. Second, per municipality, we interviewed one employee who all held a position as policy officer or policy advisor. This may have limited the diversity of perspectives and experiences within municipalities captured in this study, which may ultimately have restricted the comprehensiveness of our understanding of the local policy adoption process.

## CONCLUSION

Policy officers at Dutch municipalities demonstrated strong motivations to contribute to a smoke-free sports environment, driven by both internal and external factors. The extent and manner in which municipalities played a role in stimulating SFPs in outdoor sports clubs varied, with most municipalities fulfilling an informative and supportive role. While in some aspects, municipalities encountered challenges, they were actively using strategies to maximize their contribution to a smoke-free generation.

This study has several implications for local governments that aim to promote SFPs in outdoor sports clubs. First, it is essential to have adequate support within the municipal organization regarding SFPs at sports clubs and similar places. Second, municipalities may need to engage with various local stakeholders when promoting these SFPs. Third, financial incentives can be used to motivate sports clubs to adopt SFPs. However, further research should assess how financial incentives work within non-commercial and voluntary organizations like sports clubs.

## Supplementary Material

daae011_suppl_Supplementary_Material
