# Supplementary material for: The contribution of Dutch municipalities to stimulate smoke-free outdoor sports clubs: a qualitative study
Source: Health Promot Int. 2024 Feb 21;39(1):daae011. doi: 10.1093/heapro/daae011 (PMC10880888; doi:10.1093/heapro/daae011)
Supplement: daae011_suppl_Supplementary_Material [file daae011_suppl_supplementary_material.docx]

**Appendix A – Interview guide**

1. To what extent does your municipality play a role in stimulating outdoor sports clubs to become smoke-free?

2. What kind of activities does the municipality undertake in stimulating outdoor sports clubs to become smoke-free?

2. What were the motives for your municipality to commit itself (not/moderately/in this way) to stimulating sports clubs to become smoke-free?

3. Where there any hindering factors for your municipality to get started with stimulating outdoor sports clubs to become smoke-free and why?

4. Where there any facilitating factors for your municipality to get started with stimulating outdoor sports clubs to become smoke-free and why?

5. To what extent did sports clubs themselves play a role in the municipality's policy for smoke-free sports clubs? What kind of role did they play?

7. To what extent did the policy of other municipalities play a role in your municipality's policy for smoke-free sports clubs? What kind of role did this play?

8. To what extent did the development and/or implementation of policy aimed at other themes within your municipality play a role in the policy for smoke-free sports clubs?

9. To what extent did national policy play a role in your municipality's policy for smoke-free sports clubs? What kind of role did this play?
